# Novel Insights on Hantavirus Evolution: The Dichotomy in Evolutionary Pressures Acting on Different Hantavirus Segments

**DOI:** 10.1371/journal.pone.0133407

**Published:** 2015-07-20

**Authors:** Sathish Sankar, Mohita Upadhyay, Mageshbabu Ramamurthy, Kumaran Vadivel, Kalaiselvan Sagadevan, Balaji Nandagopal, Perumal Vivekanandan, Gopalan Sridharan

**Affiliations:** 1 Sri Sakthi Amma Institute of Biomedical Research, Sri Narayani Hospital and Research Centre, Sripuram, Vellore, 632 055, Tamil Nadu, India; 2 Kusuma School of Biological Sciences, Indian Institute of Technology, New Delhi, 110 016, India; Division of Clinical Research, UNITED STATES

## Abstract

**Background:**

Hantaviruses are important emerging zoonotic pathogens. The current understanding of hantavirus evolution is complicated by the lack of consensus on co-divergence of hantaviruses with their animal hosts. In addition, hantaviruses have long-term associations with their reservoir hosts. Analyzing the relative abundance of dinucleotides may shed new light on hantavirus evolution. We studied the relative abundance of dinucleotides and the evolutionary pressures shaping different hantavirus segments.

**Methods:**

A total of 118 sequences were analyzed; this includes 51 sequences of the S segment, 43 sequences of the M segment and 23 sequences of the L segment. The relative abundance of dinucleotides, effective codon number (ENC), codon usage biases were analyzed. Standard methods were used to investigate the relative roles of mutational pressure and translational selection on the three hantavirus segments.

**Results:**

All three segments of hantaviruses are CpG depleted. Mutational pressure is the predominant evolutionary force leading to CpG depletion among hantaviruses. Interestingly, the S segment of hantaviruses is GpU depleted and in contrast to CpG depletion, the depletion of GpU dinucleotides from the S segment is driven by translational selection. Our findings also suggest that mutational pressure is the primary evolutionary pressure acting on the S and the M segments of hantaviruses. While translational selection plays a key role in shaping the evolution of the L segment. Our findings highlight how different evolutionary pressures may contribute disproportionally to the evolution of the three hantavirus segments. These findings provide new insights on the current understanding of hantavirus evolution.

**Conclusions:**

There is a dichotomy among evolutionary pressures shaping a) the relative abundance of different dinucleotides in hantavirus genomes b) the evolution of the three hantavirus segments.

## Introduction

The relative abundance of dinucleotides and CpG dinucleotides in particularly is being increasingly studied. Studies have shown that CpG under-representation in viruses is due to mutational pressure [1, 2] or translational selection [3]. In vertebrate genomes up to 90% of CpG dinucleotides are methylated in contrast to very low levels (<5%) of CpG dinucleotides methylation among invertebrate genomes [4, 5, 6]. The cytosines within methylated CpGs are hotspots for C to T mutations [7]. As a result, CpG dinucleotides are severely depleted among vertebrate host genomes as compared to invertebrate host genomes. The most widely accepted reasons for CpG dinucleotide repression in DNA include (a) spontaneous deamination of 5-methylcytosine leads to the formation of thymine; this transition (C to T) is irreversible [8, 9] and (b) minimizing toll-like receptor 9-mediated innate immune response [10]. CpG depletion is also known to occur among RNA viruses [11]. Among all the four groups of RNA viruses, CpG dinucleotides are significantly under-represented in negative sense ssRNA viruses and retroviruses viruses [8]. While the replicative DNA intermediate step may conceivably contribute to CpG depletion among retroviruses, the exact mechanism(s) responsible for CpG depletion among negative RNA viruses is not well understood.

While most members of the family *Bunyaviridae* are transmitted by arthropod vectors, hantaviruses are not. Hantaviruses establish persistent and asymptomatic infection in rodents and insectivorous bats [12]. Hantavirus infections in humans occur primarily through human contact with an aerosolized rodent excreta [13]. Hantaviruses are important zoonotic pathogens; they cause either hemorrhagic fever with renal syndrome (HFRS) or hantavirus cardiopulmonary syndrome (HCPS) [14,15,16].

Hantaviruses are negative sense RNA viruses with a tripartite genome consisting of large (L), middle (M) and small (S) segments [15,16]. Several new genotypes of hantaviruses have been identified recently [17,18,19]. Genetic drift by neutral or quasi-neutral substitutions also plays a role in the evolution of hantaviruses [20, 21]. Evolutionary analysis suggests that rodent-borne hantaviruses probably originated 2000 years ago [22]. Initial studies suggested a slow evolution rate for hantaviruses [23]; this notion has been questioned in subsequent studies supporting high rates of molecular evolution among hantaviruses [24, 25]. Several studies have argued for [23, 26,27,28] and against [24,25] a role for co-divergence of hantaviruses with their reservoir hosts. The contribution of host-pathogen co-divergence to hantavirus evolution remains an open question, further complicating the estimates on hantavirus evolution rates. The evolutionary pressures that shape hantavirus evolution remain poorly understood.

Codon usage bias (CUB) is one of the key factors influencing virus evolution. Both translational selection and mutational pressure can influence codon usage bias. Analysis of dinucleotide frequencies and codon usage bias may provide novel insights on virus evolution [29,30]. In this study, we analyze the differences in relative abundance of dinucleotides, codon usage bias and analyse the relative roles of mutational pressure and translational selection among the three hantavirus segments. Our study will help identify the evolutionary pressures acting on hantaviruses.

## Materials and Methods

### Retrieval of sequences

The list of hantavirus species were retrieved from ICTV (International Committee on Taxonomy of Viruses,http://talk.ictvonline.org/files/ictv_documents/m/msl/5208.aspx). All full-length sequences of hantavirus genome segments (S, M and L) available in GenBank (www.ncbi.nlm.nih.gov/nucleotide) were retrieved for analysis. If more than one full-length sequences are available for a given virus, only one full-length virus sequence from a particular host was used for analysis. The virus sequences that correspond to cloned strains, cell lines or vaccines strains were excluded from analysis. A total of 118 sequences were analyzed; this includes 51 sequences of the S segment, 43 sequences of the M segment and 23 sequences of the L segment. The accession numbers of hantavirus sequences used are summarized in S1 Table.

### Calculation of dinucleotide frequencies

The observed/expected frequency for the dinucleotide (XpY) is calculated using the formula: (O/E)_XpY_ = [*f*(XY)/*f*(X) *f*(Y)]* G [2] where *f*(XY) is the frequency of the dinucleotide XpY, *f* (X) and *f*(Y) are the frequencies of mononucleotides X and Y respectively and *G* is the genome length.

### Calculation of codon usage frequencies

Codon W (http://mobyle.pasteur.fr/cgi-bin/portal.py#forms::CodonW) was used to determine the effective number of codon (ENC), GC composition. ENC values range from 20 to 61. Lower the ENC value higher the codon usage bias. The following formula: ENC* = 2+GC_3_ + {29/[(GC_3s_)^2^ + (1-GC_3s_)^2^]} was used to calculate the expected ENC value (ENC*) [31]. The influence of GC composition on codon usage bias was assessed using the ENC-GC_3_ plot [31]. In addition, the relationship between GC content at the third codon position and GC content at the non-synonymous codon positions was studied to determine the influence of translational selection and mutational pressure on virus evolution.

Relative synonymous codon usage (RSCU) is a widely used metric to assess codon usage bias among synonymous codons. If the synonymous codons of an amino acid are used with equal frequencies, the RSCU value will be one. When the RSCU value is greater than 1, the codons have positive codon usage bias and if the value of RSCU is less than 1, the codons have negative codon usage bias.

### Calculation of dinucleotide frequencies in the intracodon region

A web tool (http://www.cbs.dtu.dk/services/FeatureExtract/) was used to extract the annotated coding DNA sequences (CDS) from GenBank. The distribution of dinucleotide (XpY) in two locations intracodon region (XpY_1,2_ and XpY_2,3_) was calculated using the following formula:

(O/E)XpY_1,2_ = [*f* (X_1_Y_2_) /*f* (X_1_)*f (Y_2_)]*codon length

(O/E)XpY_2,3_ = [*f* (X_2_Y_3_) /*f* (X_2_)*f (Y_3_)]*codon length

### Statistical analysis

Statistical analysis of the data was done using Student’s *t* test and Pearson’s correlation coefficient (*r* ^*2*^). MS-Excel or Graph pad were used to make the graphs. Box plots were used to compare the distributions and correlation between parameters was evaluated using scatter plots. Results were considered statistically significant at a *P* value of <0.05.

## Results and Discussion

### Distribution of dinucleotides in hantavirus genomes

**Fig 1:**
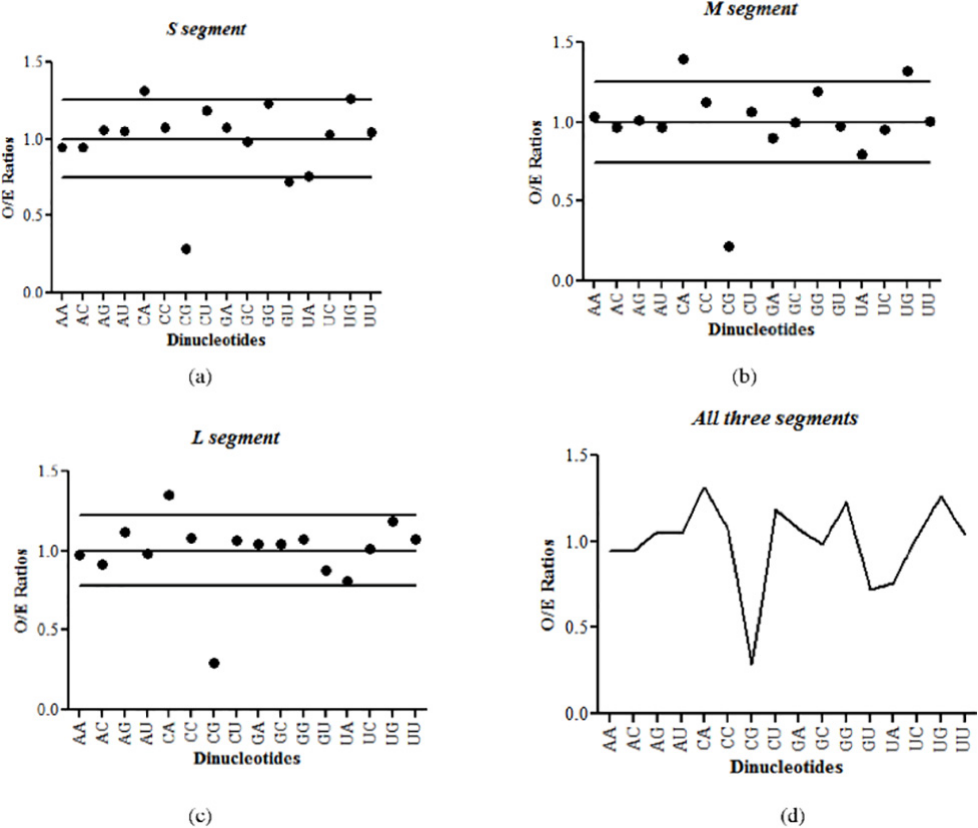
Relative abundance of dinucleotides in hantavirus genomes. (a) The mean ±standard deviation of dinucleotide O/E ratios for hantaviruses S segment is 1.0±0.25. The O/E ratios for most of the dinucleotides lie inside the confidence interval of 0.75–1.25 with the exceptions of CpG and GpU dinucleotides (underrepresented) and CpA dinucleotides (overrepresented). (b) The mean±standard deviation of dinucleotide O/E ratios for hantaviruses M segment is 1.0±0.25. CpG dinucleotides were underrepresented and CpA and UpG dinucleotides were overrepresented as the O/E ratios for these dinucleotides were located outside the confidence interval of 0.75–1.25. (c) The mean ±standard deviation of dinucleotide O/E ratios for hantaviruses L segment is 1.0±0.22. CpG and CpA dinucleotides were the most deviant dinucleotides as their O/E ratios were located outside the confidence interval of 0.78–1.22. (d) Comparison of the average dinucleotide O/E ratios for all three segments of hantaviruses. The depletion of CpG dinucleotides was common across all the three segments of hantaviruses.

We analyzed the frequencies of all 16 dinucleotides for 51 S segments, 43 M segments and 24 L segments of hantaviruses. The relative abundance of dinucleotides in the three segments (S, M, L) of hantaviruses is shown in Fig 1. The mean ±standard deviation of dinucleotide O/E ratios for hantaviruses S-, M- and L-segments are 1.0±0.25, 1.0±0.25, 1.0±0.22 respectively (Fig 1A, 1B and 1C). CpG dinucleotides were found to be the most depleted dinucleotides in all the three segments of hantaviruses as compared to any other dinucleotide (Fig 1D; P<0.0001). CpA and UpG over-representation and UpA depletion were seen in all three segments. CpA and UpG over-representation has been observed to occur concomitantly with CpG depletion in both DNA [2] and RNA virus genomes [11]. UpA depletion is a universal feature of animal [32] and microbial genomes [33]. Universal UpA depletion has been linked to increased sensitivity of the UpA dinucleotides to ribonucleases [34]. In addition we also found significant GpU depletion in the S segment of hantaviruses.

### Hantavirus genomes are depleted for CpG dinucleotides

**Fig 2:**
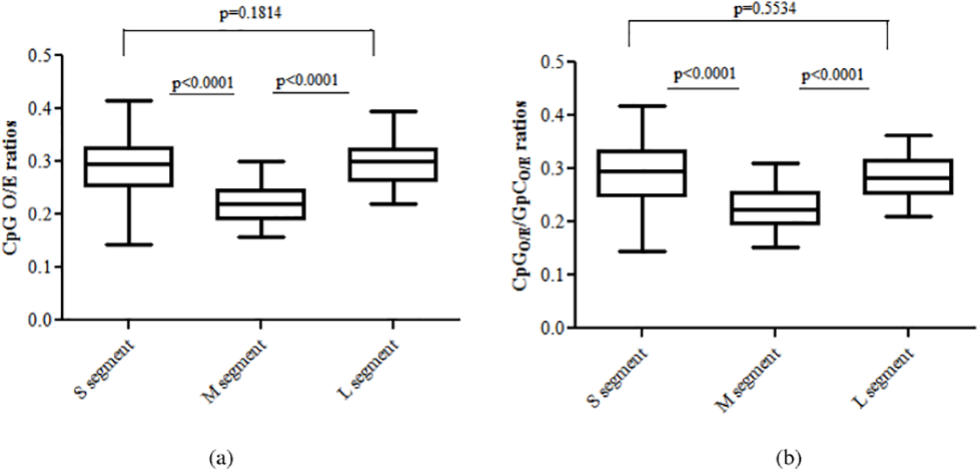
Hantavirus genomes are depleted for CpG dinucleotides and underlying evolutionary pressure is specific to CpG (not GpC) dinucleotides. (a) Among the 3 segments of hantaviruses the CpG dinucleotide O/E ratios for the M segment were significantly lower than that for the S segment (0.22±0.04 vs 0.28±0.06; P<0.0001) and that for the L segment (0.22±0.04 vs 0.29±0.05; P<0.0001). The differences in CpG O/E ratios of the S segment and the L segment were not significant (0.28±0.06 vs 0.29±0.05; P = 0.18). (b) The CpG/GpC O/E ratios were significantly lower for the M segment of hantaviruses as compared to those for the S segment (0.22±0.04 vs 0.29±0.07; P<0.0001) and for the L segment (0.22±0.04 vs 0.28±0.04; P<0.0001); clearly demonstrating that CpG dinucleotides but not GpC dinucleotides are susceptible to the underlying evolutionary pressures.

The depletion of CpG dinucleotides was common across all the three segments of hantaviruses (Fig 1A, 1B and 1C). Within the 3 segments of hantaviruses, the CpG dinucleotide O/E ratios for the M segment were significantly lower than that for the S segment (0.22±0.04 vs 0.28±0.06; P<0.0001; Fig 2A) and that for the L segment (0.22±0.04 vs 0.29±0.05; P<0.0001; Fig 2A). The differences in CpG O/E ratios of the S segment and the L segment were not significant (0.28±0.06 vs 0.29±0.05; P = 0.18; Fig 2A). CpG dinucleotide depletion is the most well-studied dinucleotide variation among vertebrate DNA viruses [35], RNA viruses [11] and single-stranded DNA viruses [2]. Nonetheless, this is the first report on CpG depletion among the three hantavirus segments.

CpG and GpC dinucleotides contain the same mononucleotides. To confirm that the CpG depletion is not due to pressures acting on the constituent mononucleotides (C and G) we assessed the CpG/GpC O/E ratios. The CpG/GpC O/E ratios were significantly lower for the M segment of hantaviruses as compared to those for the S segment (0.22±0.04 vs 0.29±0.07; P<0.0001) and for the L segment (0.22±0.04 vs 0.28±0.04; P<0.0001); demonstrating that CpG dinucleotides but not GpC dinucleotides are susceptible to evolutionary pressures (Fig 2B).

### Avoidance of CpG-containing codons in all three segments of hantaviruses

**Fig 3:**
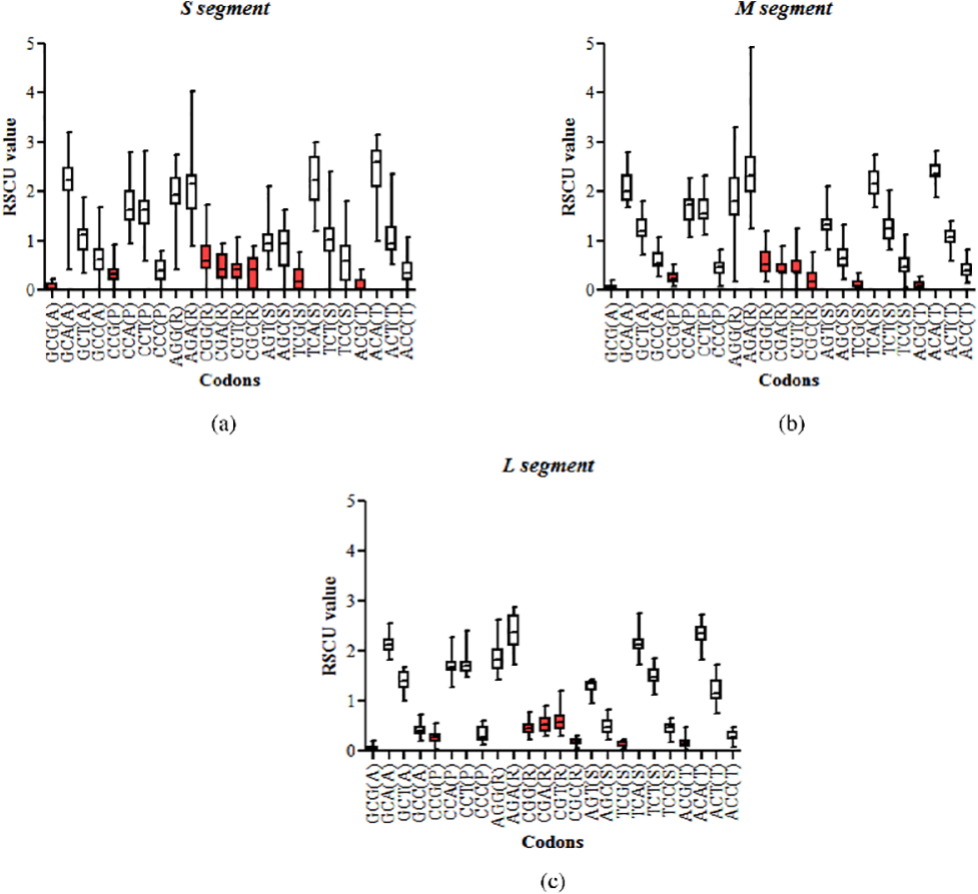
Avoidance of CpG-containing codons in all three segments of hantaviruses. Box plots with RSCU values of synonymous CpG-containing codons. Data for CpG-containing codons are shown in red boxes. The single letter code for amino acids is indicated within parenthesis adjacent to the codons. The median RSCU values of all CpG-containing codons were less than 1 in all the 3 segments, clearly suggesting that CpG-containing codons were avoided in hantavirus genomes.

We then investigated if the genome-wide depletion of CpG dinucleotides influenced the usage of CpG-containing codons. Preferentially used codons have relative synonymous codon usage (RSCU) values greater than one; codons used sub-optimally have RSCU values less than one. In order to understand the influence of genome-wide CpG dinucleotide depletion on synonymous codon usage preferences in hantavirus genomes, RSCU values of synonymous CpG-containing codons were analysed (Fig 3).

All the three segments of hantavirus strongly avoided CpG-containing synonymous codons. The RSCU values of all CpG-containing codons were less than 1 in all the 3 segments, clearly suggesting that CpG-containing codons were avoided in hantavirus genomes (Fig 3A, 3B and 3C). The average RSCU values for CpG-containing codons were similar between M segment and S segment (0.28±0.20 vs 0.34±0.20; P = 0.564) or between M and L segment (0.28±0.20 vs 0.30±0.20; P = 0.872).

The data suggest that CpG depletion, being the most pronounced dinucleotide variation among hantaviruses, plays a key role in the evolution of this group of viruses. Intrigued by major depletion of CpG dinucleotides among hantaviruses, we went on to investigate possible underlying evolutionary pressures leading to CpG depletion.

### Mutational pressure leads to CpG dinucleotide depletion in hantaviruses

In order to understand the relative roles of mutational pressure and translational selection in leading to CpG depletion among hantaviruses we analyzed to difference between intracodon dinucleotide O/E ratios (i.e. XpY_1,2_ and XpY_2,3_ for the dinucleotide XpY) and the genome-wide dinucleotide O/E ratios for a given dinucleotide. For example, we calculated the difference between genome-wide CpG O/E ratio and the average intracodon CpG O/E ratio [i.e average of CpG O/E ratio at the first-second position (CpG_1,2_ or CGN) and the second-third codon position (CpG_2,3_ or NCG)] for each segment. If mutational pressure drives the depletion of CpGs, it is likely that the intracodon CpG O/E ratios will be higher or same as the genome-wide CpG O/E ratios (i.e. the depletion of CpG dinucleotides is more pronounced throughout the genome than within the intracodon region). On the contrary, if the CpG depletion is primarily driven by translational selection, then the depletion of CpG within the intracodon regions will be more pronounced as compared to that within the whole genome (i.e intracodon CpG O/E ratio would be lower than the genome-wide CpG O/E ratio).

**Fig 4:**
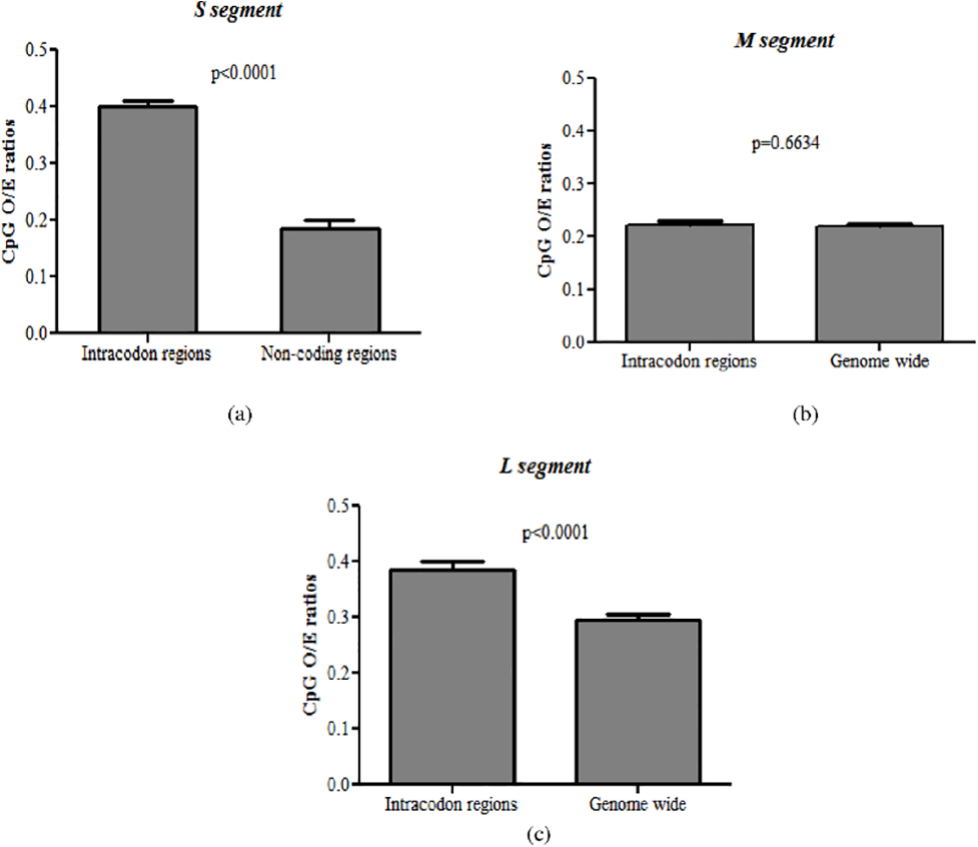
Mutational pressure leads to CpG dinucleotide depletion in hantaviruses. (a) CpG depletion was more pronounced in the non-coding region of the S segment as compared to that within the codons (intracodon CpG O/E); clearly suggesting that mutational pressure is the major evolutionary force leading to the loss of CpG dinucleotides in the S segment of hantaviruses. (b) The genome-wide CpG O/E ratios were same as the intracodon CpG O/E ratios for the M segment (0.22±0.04 vs 0.22±0.04; P = 0.663) (c) The genome-wide CpG O/E ratios were significantly lower than intracodon CpG O/E ratios (0.29±0.05 vs 0.39±0.07; P<0.0001; Fig 4c) for L segment. These findings clearly suggest that the CpG depletion in S, M and L segments are driven by genome-wide mutational pressure and not translational selection.

The genome-wide CpG O/E ratios were same as the intracodon CpG O/E ratios for the M segment (0.22±0.04 vs 0.22±0.04; P = 0.663; Fig 4B) and for L segment the genome-wide CpG O/E ratios were significantly lower than intracodon CpG O/E ratios (0.29±0.05vs 0.39±0.07; P<0.0001; Fig 4C); this finding clearly suggests that the CpG depletion in M and L segments are driven by genome-wide mutational pressure and not translational selection. Unlike the M and the L segments in which most of the genome is constituted by intracodon region, a considerable portion (>25%) of the hantavirus S segment represents non-intracodon region. Therefore, for the S segment we analyzed the CpG O/E ratios for the non-coding region (varying in length from 168 to 685 nucleotides) and within the codons (intracodon CpG O/E). If translational selection predominates over mutational pressure as the cause of CpG dinucleotide depletion, one would expect that the CpG depletion within the codons (intracodon CpG O/E) is more pronounced than that in the non-coding region. Our analysis indicates that CpG depletion is more pronounced in the non-coding region of the S segment as compared that within the codons (intracodon CpG O/E) (0.19±0.09 vs 0.40± 0.08; P<0.0001; Fig 4A); this analysis supports the notion that mutational pressure is the major evolutionary force leading to the loss of CpG dinucleotides in the S segment of hantaviruses.

Avoidance of CpG-containing synonymous codons or translational selection of non-CpG containing codons has been linked to the loss of CpG dinucleotides in RNA viruses infecting vertebrates, invertebrates, plants, bacteria and fungi [8]. In contrast, our findings suggest that depletion of CpG dinucleotides in all three segments of hantaviruses is linked to mutational pressure.

### Hantavirus S segments are GpU depleted

**Fig 5:**
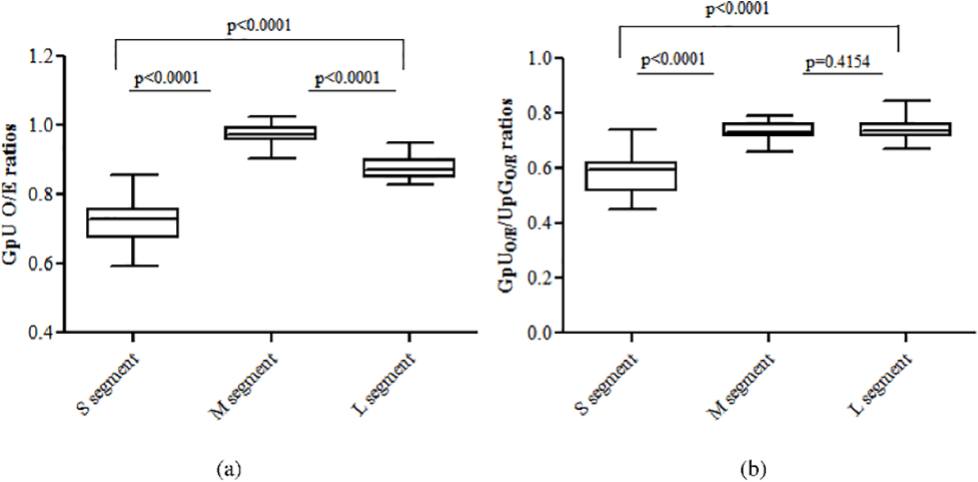
Hantavirus S segments are GpU depleted and that underlying evolutionary pressure is specific to GpU (not UpG) dinucleotides. (a) The hantavirus S segment had significantly lower GpU dinucleotide O/E ratios as compared to that in the M segment (0.72±0.06 vs 0.97±0.03; P<0.0001) or the L segment (0.72±0.06 vs 0.86±0.04; P<0.0001). (b) The GpU/UpG O/E ratios were found to be significantly lower in hantaviruses for the S segment as compared to those for the M segment (0.58±0.07 vs 0.74±0.03; P<0.0001) or for the L segment (0.58±0.07 vs 0.74±0.05; P<0.0001); suggesting that GpU depletion in the S segment is not linked to pressures acting on the constituent mononucleotides (G or U) but is due to pressures specifically acting on GpU dinucleotides.

Interestingly, we also observed major GpU dinucleotide depletion in the S segment. The hantavirus S segment has significantly lower GpU dinucleotide O/E ratios as compared to that in the M segment (0.72±0.06 vs 0.97±0.03; P<0.0001; Fig 5A) or the L segment (0.72±0.06 vs 0.86±0.04; P<0.0001; Fig 5A). The GpU/UpG O/E ratios were found to be significantly lower in hantaviruses for the S segment as compared to those for the M segment (0.58±0.07 vs 0.74±0.03;P<0.0001; Fig 5B) or for the L segment (0.58±0.07 vs 0.74±0.05; P<0.0001; Fig 5B). This finding suggests that GpU depletion in the S segment is not linked to pressures acting on the constituent mononucleotides (G or U) but is due to pressures specifically acting on GpU dinucleotides.

The depletion of GpU has been reported in the human genome and mitochondrial genomes [36]. In addition, based on analysis of about 45 million SNPs, Simmonds *et al*., identified GpU as one of the most mutable dinucleotides in humans and other mammals [1]. To our knowledge, GpU depletion has not been reported in virus genomes. The GpU depleted S segment encodes the hantavirus nucleoprotein, a multifunctional protein which interacts with the hantavirus polymerase and a hantavirus glycoprotein [37]. In addition, the hantavirus nucleoprotein interferes with key regulatory host proteins in the host cells [38].

### Avoidance of GpU-containing codons in the S segment of hantaviruses

We investigated if the GpU depletion in the S segment influenced the usage of GpU-containing synonymous codons. GpU-containing synonymous codons encoding valine (GUU, GUC, GUA and GUG) were excluded as all the four synonymous codons contain GpU and hence will not allow meaningful interpretation. This left us with only glycine (GGU, GGC, GGA, GGG) among GpU-containing 3-fold or 4-fold degenerate codons. In addition, we also excluded the GpU-containing synonymous codons CGU (Arg) and AGU (Ser) from the analysis since they contain both CpG and GpU dinucleotides or other synonymous codons contain CpG dinucleotides. Since all the three segments were found to display a strong bias against CpG-containing codons we had to exclude GpU containing synonymous codons for arginine and serine.

**Fig 6:**
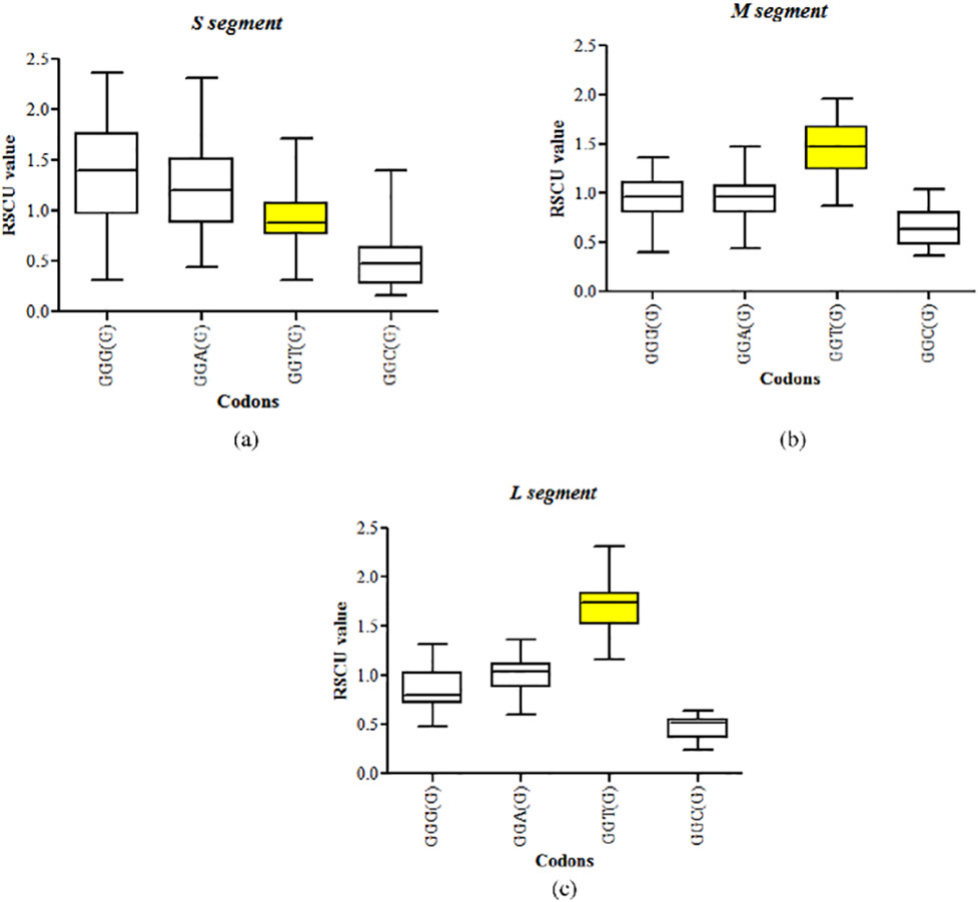
Avoidance of GpU-containing codons in the S segment of hantaviruses. Box plots showing the RSCU values of synonymous GpU-containing codons. The RSCU values of GpU-containing codons are shown in yellow boxes. GpU-containing codons were avoided in the S segment of hantaviruses as evidenced by median RSCU values of less than one. The average RSCU values for GpT-containing codons in the S segment were lower as compared those in the M segment (0.91±0.32 vs 1.46±0.25; P<0.0001) or the L segment (0.91±0.32 vs 1.7±0.27; P<0.0001); this is a reflection of GpU dinucleotides depletion from the S segment.

GpU-containing codons were avoided in the S segment of hantaviruses as evidenced by RSCU values of less than one (Fig 6). The average RSCU values for GpU-containing codons in the S segment were lower as compared those in the M segment (0.91±0.32 vs 1.46±0.25; P<0.0001) or the L segment (0.91±0.32 vs 1.7±0.27; P<0.0001); this is a reflection of GpU dinucleotides depletion from the S segment.

### GpU dinucleotide depletion in the S segment is linked to translational selection

**Fig 7:**
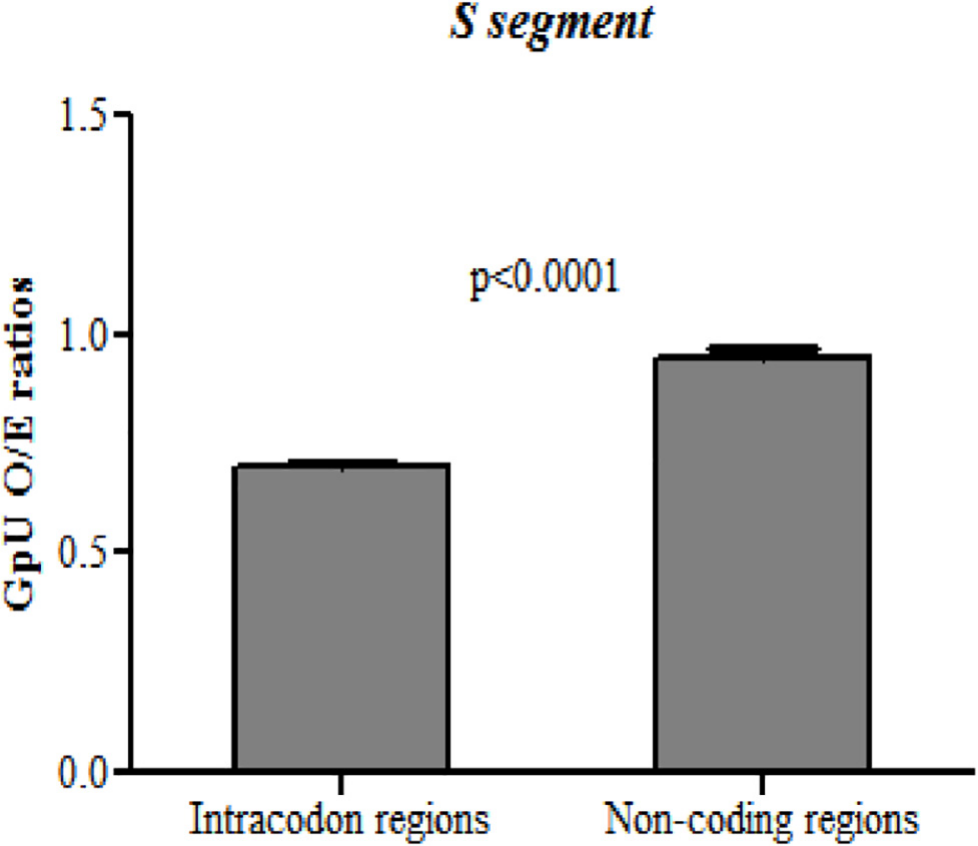
GpU dinucleotide depletion in the S segment is linked to translational selection. The intracodon GpU O/E ratio for the S segment was significantly lower than that for the non-coding region of this segment (0.70±0.07 vs 0.95±0.14; P< 0.0001); clearly supporting translational selection as the major driver of GpU depletion in the S segment of hantaviruses.

We analyzed the GpU O/E ratios for the non-coding region and the coding region (intracodon O/E ratio) of the S segment. We found that the intracodon GpU O/E ratio for the S segment was significantly lower than that for the non-coding region of this segment (0.70±0.07 vs 0.95±0.14; P<0.0001; Fig 7); clearly supporting translational selection as the major driver of GpU depletion in the S segment of hantaviruses. This finding is in contrast to our findings that mutational pressure is the primary evolutionary force leading to the depletion of CpG dinucleotides in all the three segments of hantaviruses. Our results suggest that the relative abundance of different dinucleotides within the S segment may be driven by different evolutionary forces. Nonetheless, it is not clear why GpU depletion is restricted to the S segment of hantaviruses.

### CpG dinucleotide depletion in the M segment is linked to evolutionary lineage of host

**Fig 8:**
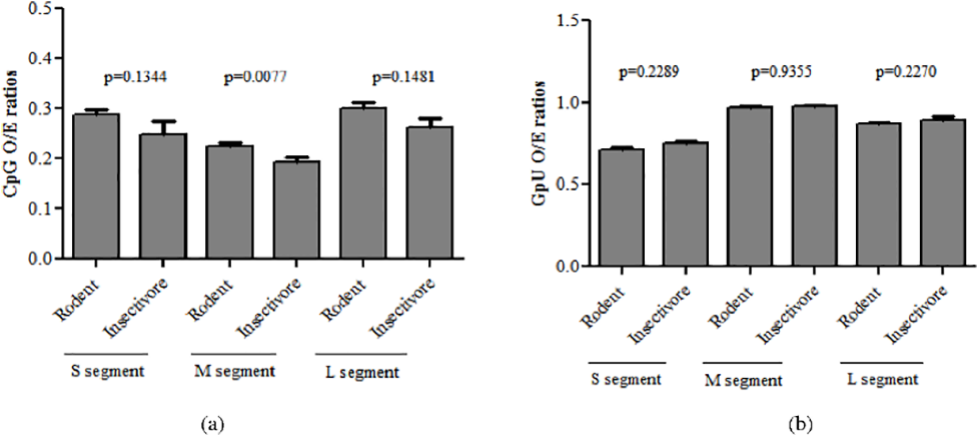
CpG dinucleotide depletion in the M segment is linked to evolutionary lineage of host. (a) In M segment the CpG O/E ratios were significantly lower in insectivore borne viruses than that in rodent borne viruses (0.19±0.03 vs 0.22±0.03; P = 0.0077). The average CpG O/E ratios were lower in insectivore borne hantaviruses as compared to rodent borne hantaviruses for S segment (0.25±0.08 vs 0.29±0.06; P = 0.1344) and L segment (0.26±0.03 vs 0.30±0.05; P = 0.1481); however the difference was not statistically significant. (b) The GpU O/E ratios was not statistically different between insectivore borne and rodent borne hantaviruses in S segment (0.75±0.06 vs 0.72±0.06; P = 0.2289), M segment (0.97±0.02 vs 0.97±0.03;P = 0.9355) and L segment (0.89±0.05 vs 0.87±0.03).

After having demonstrated that the depletion of CpG dinucleotides in M segment is linked to mutational pressure and depletion of GpU dinucleotide in S segment is linked to translational selection, we investigated differences, if any in analyzed CpG and GpU O/E ratios between rodent-borne hantaviruses and insectivore-borne hantaviruses across the three segments. Interestingly, CpG O/E ratios for the M were significantly lower in insectivore borne hantaviruses as compared to that in rodent-borne hantaviruses (0.19±0.03 vs 0.22±0.03; P = 0.007; Fig 8A). Increased CpG dinucleotide depletion in the M segment of insectivore-borne hantaviruses as compared to rodent-borne hantaviruses is particularly interesting given that (a) Insectivores are more closely related to mammals than are rodents [39] and (b) The link between the evolutionary lineage of the animal genome and the extent of CpG dinucleotide depletion [5]. For example, human genome is more CpG depleted than most vertebrate genomes. Increased CpG dinucleotide depletion among animal genomes [5 *]*. It is therefore possible that increased CpG depletion in the M segment of insectivore-borne hantaviruses as compared to rodent-borne hantaviruses may reflect co-evolution with the host. The average CpG O/E ratios among the insectivore-borne hantaviruses were marginally lower as compared to that among the rodent-borne hantaviruses for both the S segment (0.25±0.08 vs 0.29±0.06; P = 0.1344) and the L segment (0.26±0.03 vs 0.30±0.05; P = 0.1481; Fig 7A); however, these differences were not statistically significant. Our findings clear demonstrate that CpG depletion is most pronounced in the hantavirus M segment and differences in CpG O/E ratios do exist between insectivore-borne hantaviruses and rodent-borne hantaviruses in the M segment. However, our results do not address why CpG O/E ratios in the S and L segment are comparable between insectivore-borne hantaviruses and rodent-borne hantaviruses.

The GpU O/E ratios were comparable between insectivore-borne and rodent-borne hantaviruses in the S segment (0.75±0.06 vs 0.72±0.06; P = 0.2289), M segment (0.97±0.02 vs 0.97±0.03; P = 0.9355) and L segment (0.89±0.05 vs 0.87±0.03) (Fig 8B).

### Differences in the evolutionary forces shaping the three hantavirus segments

We then sought to investigate if there are differences in the relative roles of mutational pressures and translational selection in shaping the evolution of the three hantavirus segments. We therefore analyzed the relationship between GC_3_ and GC_1,2_ and also codon usage bias among the three hantavirus segments.

**Fig 9:**
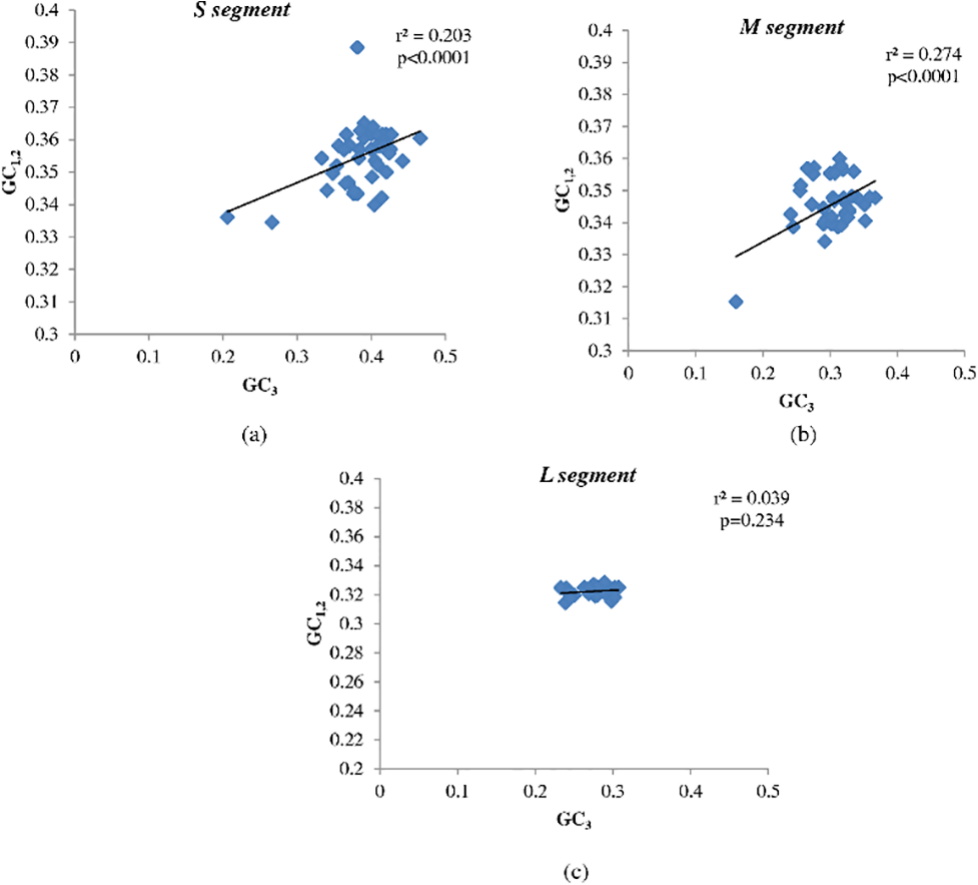
Neutrality plot. Scatter plot demonstrating significant correlation between GC_3_ (X-axis) and GC_1,2_ (Y-axis) among (a) S segment (*r* ^2^ = 0.203, P<0.0001) and (b) M segment (*r* ^2^ = 0.274, P<0.0001), implying a major role for mutational pressure (and not translational selection) in the evolution of the S and the M segments. (c) There was no correlation between GC_3_ and GC_1,2_ in the L segment (*r* ^2^ = 0.039, P = 0.234) suggesting that translational selection may play an important role in the evolution of this segment.

The lack of correlation or a weak correlation between GC_3_ and GC_1,2_ would suggest a major role of translational selection (as translational selection has a dissimilar influence on the three positions within the codon); while a strong correlation between GC_3_ and GC_1,2_ would support a key role for mutational pressure (all the codon positions are equally affected). In our study, we found significant correlation between GC_3_ and GC_1,2_ in the S and M segments of hantaviruses (S segment: *r* ^2^ = 0.203, P<0.0001, Fig 9A; M segment: *r* ^2^ = 0.274, P<0.0001, Fig 9B), implying a major role for mutational pressure (and not translational selection) in the evolution of the S and the M segments. The absence of major translational selection / pronounced codon usage bias may facilitate hantavirus replication in humans, rodents and non-rodent mammalian hosts.

In contrast, there was no correlation between GC_3_ and GC_1,2_ in the L segment (*r* ^2^ = 0.039, P = 0.234, Fig 9CV) suggesting that translational selection may play an important role in the evolution of this segment that encodes the viral polymerase. Neutrality plots (correlation between GC_3_ and GC_1,2_) suggest that different evolutionary pressures may predominate among hantavirus segments.

Additional evidence supporting the dichotomy in evolutionary pressures shaping different hantavirus segments comes from analysis of codon usage bias. To examine the differences in overall codon usage bias among the three segments, ENC (effective number of codons) was used as an index [31,40]. The ENC values ranged from 41.24 to 55.13 (mean±SD: 50.11±2.52) in the S segment of hantaviruses, and from 37 to 52.39 for the M segment (mean±SD: 47.93±3.11) and from 42.38 to 47.60 in L segment (mean±SD: 45.49±1.52).

**Fig 10:**
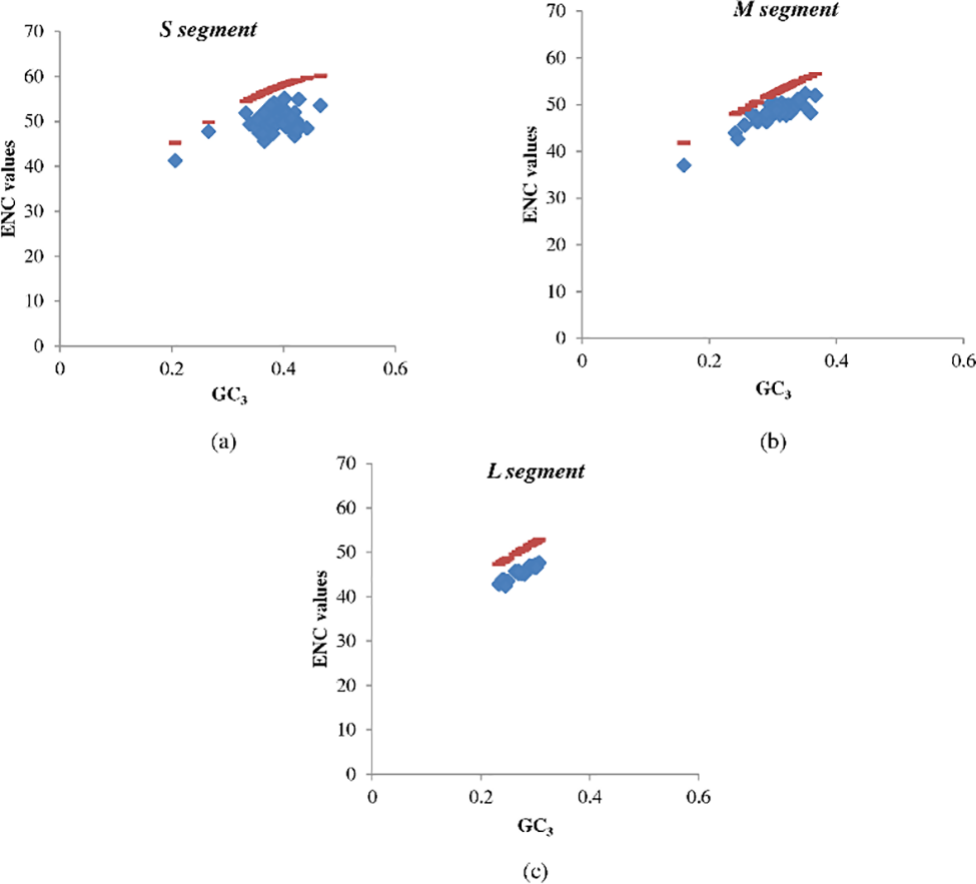
ENC-GC_3_ plot. Correlation between GC_3_ and the effective codon usage statistic (ENC) among (a) S segment, (b) M segment and (c) L segment. The red line represents the ENC expected values (ENC*) and the ENC values are shown in blue. ENC values for the L segment are significantly lower than that for the S segment (45.49±1.52 vs 50.11±2.52; P<0.0001) or the M segment (45.49±1.52 vs 47.93±3.11; P = 0.0003), suggesting that mutational pressure is the predominant evolutionary force acting on the S and M segments; while translational selection predominates in the evolution of the L segment.

The relationship between GC content at the third codon position (GC_3_) and ENC was examined using the ENC-GC_3_ plot. The ENC-GC_3_ plot is used to study the influence of mutational pressure or translational selection on evolution (Fig 10). We found that ENC values for the L segment were significantly lower than that for the S segment (45.49±1.52 vs 50.11±2.52; P<0.0001) or the M segment (45.49±1.52 vs 47.93±3.11; P = 0.0006). Taken together, these findings unequivocally demonstrate that mutational pressure is the predominant evolutionary force acting on S and M segments; while translational selection predominates as the major overall evolutionary force in the L segment. To our knowledge, this is the first report highlighting the role of distinct evolutionary pressures in shaping the evolution of different segments of a given virus.

In our study, the emergence of translational selection as the predominant evolutionary force underlying the evolution of the L segment is particularly interesting considering that a) RNA viruses mutate at a very high rate [41] and b) mutational pressure is in general believed to play a major role in the evolution of RNA viruses [42]. Our findings of increased translational selection in the hantavirus polymerase-encoding L segment are in keeping with a previous report on increased codon usage bias in virus polymerase-encoding region among RNA viruses as compared to more abundantly expressed structural proteins [42]. It is not clear why the more immunogenic nucleocapsid and glycoproteins are subjected to similar translational selection. The precise reasons for increased codon usage bias or translational selection in virus-polymerase encoding region merits further investigation.

A study analyzing the co-divergence of hantaviruses found more clade-defining amino acids in the L segment of hantavirus genome than in the S- or the M-segment [25]; the authors argue that the adaptive evolution of the polymerase gene may have facilitated the jump from the old world to new world rodents among hantaviruses. Another study analyzing the adaptation of puumala hantavirus to vero E6 cells found amino-acid substitutions in the coding sequence of the L segment but not within that of the S or the M segment [43]. In addition, a previous report suggests that amino acid differences in the hantavirus polymerase (encoded by the L segment) influence the host range and virulence of this group of viruses [44]. Our finding that translational selection is the key player in the evolution of L segment is in keeping with the presence of higher number of clade-defining amino acids in the L segment and its potential role in host-switching. Translational selection may be particularly important during transmission of hantaviruses among closely related hosts. It is possible that the compatibility of the L-segment encoded hantavirus polymerase may limit cross-species transmission. Nonetheless, a cross-species transmission event may specifically contribute to the evolution of polymerase by translational selection in the new host.

Both mutational pressure and translational selection may represent host-induced evolutionary forces that conceivably impact virus evolution. Nonetheless, the dichotomy in evolutionary pressures shaping the three hantavirus segments has noteworthy implications: a) While mutational pressure will influence the coding and non-intracodon region alike, translational selection will selectively influence only the intracodon region. b) A nucleotide substitution due to mutational pressure in the non-coding region is more likely to become fixed in the virus genome as compared to that in the coding region due to constraints on encoding a functional protein. In other words, mutational pressure driven evolution is likely to have a more pronounced effect on the evolution of the non-coding region of the genome. This may be particularly important for the evolution of the S segment that has a sizable non-coding region. c) While there are no major documented differences among host-induced mutational pressures among hantavirus animal hosts, the diversity of t-RNA species among higher eukaryotes [45] may potentially influence translational selection; this may influence host-specific adaptation of hantaviruses, particularly the L segment that is more amenable to translational selection.

Understanding the evolution of hantaviruses has been particularly challenging due to a) the ability of hantaviruses to infect multiple host species b) evidence supporting reassortment among hantaviruses c) studies arguing for and against host-hantavirus co-divergence and d) uncertainties on the estimates of hantavirus mutation rates. We report CpG depletion among all hantavirus segments; this is particularly pronounced in the M segment. The depletion of CpG dinucleotides among hantaviruses is primarily driven by mutational pressure. In contrast, the loss of GpU dinucleotides from hantavirus S segments is linked to translational selection against GpU-containing codons within the coding region of this segment. Our findings show for the first time that dissimilar evolutionary pressures may determine the relative abundance of different dinucleotides within virus genomes. In addition, our findings clearly indicate that the evolution of S and the M segments of hantaviruses is primarily driven by mutational pressure and not translational selection. While translational selection is the major force shaping the evolution of the L segment. Our study reveals the dichotomy in evolutionary pressures shaping the evolution of different hantavirus segments. Our findings suggest that different evolutionary pressures may contribute disproportionally to the evolution of different segments of a given virus. In sum, this study provides a new perspective on the fundamental evolutionary pressures shaping the evolution of different hantavirus segments.

## Supporting Information

S1 Table Accession numbers of hantavirus sequences analysed. (XLSX)
